# All Roads Lead to Cytosol: *Trypanosoma cruzi* Multi-Strategic Approach to Invasion

**DOI:** 10.3389/fcimb.2021.634793

**Published:** 2021-03-05

**Authors:** Gabriel Ferri, Martin M. Edreira

**Affiliations:** ^1^CONICET-Universidad de Buenos Aires, IQUIBICEN, Ciudad de Buenos Aires, Argentina; ^2^Laboratorio de Biología Molecular de Trypanosoma, Departamento de Química Biológica, Facultad de Ciencias Exactas y Naturales, Universidad de Buenos, Ciudad de Buenos Aires, Argentina; ^3^Department of Pharmacology and Chemical Biology, School of Medicine, University of Pittsburgh, Pittsburgh, PA, United States

**Keywords:** invasion, internalization, lysosome-mediated invasion, exocytic pathway, autophagic pathway, host signaling, host/parasite interaction

## Abstract

*T. cruzi* has a complex life cycle involving four developmental stages namely, epimastigotes, metacyclic trypomastigotes, amastigotes and bloodstream trypomastigotes. Although trypomastigotes are the infective forms, extracellular amastigotes have also shown the ability to invade host cells. Both stages can invade a broad spectrum of host tissues, in fact, almost any nucleated cell can be the target of infection. To add complexity, the parasite presents high genetic variability with differential characteristics such as infectivity. In this review, we address the several strategies *T. cruzi* has developed to subvert the host cell signaling machinery in order to gain access to the host cell cytoplasm. Special attention is made to the numerous parasite/host protein interactions and to the set of signaling cascades activated during the formation of a parasite-containing vesicle, the parasitophorous vacuole, from which the parasite escapes to the cytosol, where differentiation and replication take place.

## Introduction

Chagas Disease is a serious life-threatening disease caused by the protozoan parasite *Trypanosoma cruzi* and transmitted by blood-sucking triatomine insects from the Reduviidae family. In addition to an estimated of 6–8 million infected people and an alarming 50,000 deaths per year, 65–100 million people are living in areas at risk for infection (Lidani et al., 2019). This regional issue is now becoming global due to the migration of infected people to non-endemic countries, resulting in an estimated global economic burden of $7.19 billion (Lee et al., 2013).

*T. cruzi* has a complex life cycle, involving an insect vector and a mammalian host. Typically, metacyclic trypomastigotes (MTs) gain access to the mammalian host through feces contamination at the insect bite wound. Upon internalization by the host cells close to the site of entry, MTs initially reside in a parasite-containing vesicle, the parasitophorous vacuole (TcPV), from which they escape to the host cell cytoplasm and differentiate into the proliferative amastigote form. After several rounds of replication, amastigotes differentiate into motile flagellated trypomastigotes, bloodstream (BSTs), or tissue culture-derived (TCTs) trypomastigotes, that are released into the bloodstream, from where they could disseminate by infecting distant tissues or taken up by the triatomine vector during a bloodmeal (Monteón et al., 1996). Interestingly, although the parasite could potentially infect any nucleated cells, it has been demonstrated that different strains exhibit distinct tropism, measured as parasite load, for organs such as esophagus, liver, spleen, intestine, heart, and skeletal muscle, during acute phase of the infection, while tropism in the chronic phase has shown to be more homogeneous and restricted to intestine, skeletal muscle, and heart (Santi-Rocca et al., 2017). In this regard, it is worth to mention that adipocytes are also an important target cell during the acute phase of the disease, and may represent an important long-term reservoir for parasites during chronic infection (Combs et al., 2005). Additionally, it has been shown that amastigotes represent 10% of the parasites circulating in the blood of infected animals during the acute phase of infection (Andrews et al., 1987). Extracellular amastigotes (EAs), originated from premature rupture of infected cells or transformed from BSTs, are also infective and can disseminate in the infected hosts (Walker et al., 2014; Bonfim-Melo et al., 2018a).

In addition to a complex life cycle, *T. cruzi* has shown to be a remarkably heterogeneous taxon, that presents multiple strains with a high degree of genetic variability. This immense genetic diversity has been classified into six Discrete Typing Units (DTUs): the ancestral strains DTU-I and II, homozygote-derived hybrids DTU-III and IV, and heterozygote hybrids DTU-V and VI (Zingales et al., 2009). *T. cruzi’s* genome presents a conserved core of genes and extremely variable multigene surface proteins families (Berná et al., 2018). These multigene families are expanded in the genome, accordingly to its repetitive structure, and there is a rich source of diversity between different strains (De Pablos and Osuna, 2012). Among these families are the Trans-sialidase (TS) superfamily (about 1400 genes), the mucin family (about 860 genes), the Dispersed Gene Family-1 (DGF-1) family (565 genes) and the Mucin-Associated Surface Proteins (MASPs) family, which comprises around 1370 genes (El-Sayed et al., 2005; Kawashita et al., 2009). This incredible number of genes, coupled to tightly regulated post-transcriptional control of gene expression, are key players in the specific stage expression of the main surface constituents (Herreros-Cabello et al., 2020). As a consequence of the great expansion of surface protein families, the parasite is able to interact with a large number of surface receptors on the different host cells, a fundamental requirement for invasion.

In the process of invasion, the parasite hijacks the host cellular functions with the ultimate goal of establishing the replicative niche. Several pathways, converging in the formation of the TcPV, have been implicated in host cell invasion (Barrias et al., 2013). In general, *T. cruzi* invasion can be divided into four major steps: 1) host cell recognition and adhesion, 2) parasite internalization, 3) TcPVformation and maturation, and 4) escape to the cytosol. In this review, we highlight the different host cell signaling pathways that the parasite exploits to promote internalization, TcPV formation and the establishment of a productive intracellular infection.

The focus will be first placed on three strategies that *T. cruzi* uses to hijack host cell signaling pathways to facilitate invasion: 1) Engagement of host cell surface receptors (Alba Soto and González Cappa, 2019); 2) Protein and molecule shedding, including microvesicles and other vesicles, such as exosomes (Borges et al., 2016; Watanabe Costa et al., 2016); and 3) Host cell plasma membrane mechanical wounding (Fernandes and Andrews, 2012). These events converge in preparing the cell for subsequent invasion. The display of redundant strategies is crucial because it guarantees an effective invasion by *T. cruzi*.

Second, attention will be placed on the strategies that lead to the internalization of the parasite. *T. cruzi* exploits three main mechanisms in the host cell to facilitate internalization: a) Ca^2+^-dependent recruitment of lysosomes, b) Endocytosis, and c) Autophagy. As a result of the activation of these pathways, invading trypomastigotes end up localizing inside the TcPV. The mechanism for vacuolar escape is known to be lysosome- and pH- dependent, involving secretion of a porin-like/complement 9-related factor TcTOX (Andrews, 1994). As an obligate intracellular parasite, ensuring cell integrity is essential for the establishment of a productive infection. Accordingly, signaling pathways are also manipulated to avoid apoptosis (Stahl et al., 2014).

**Figure 1:**
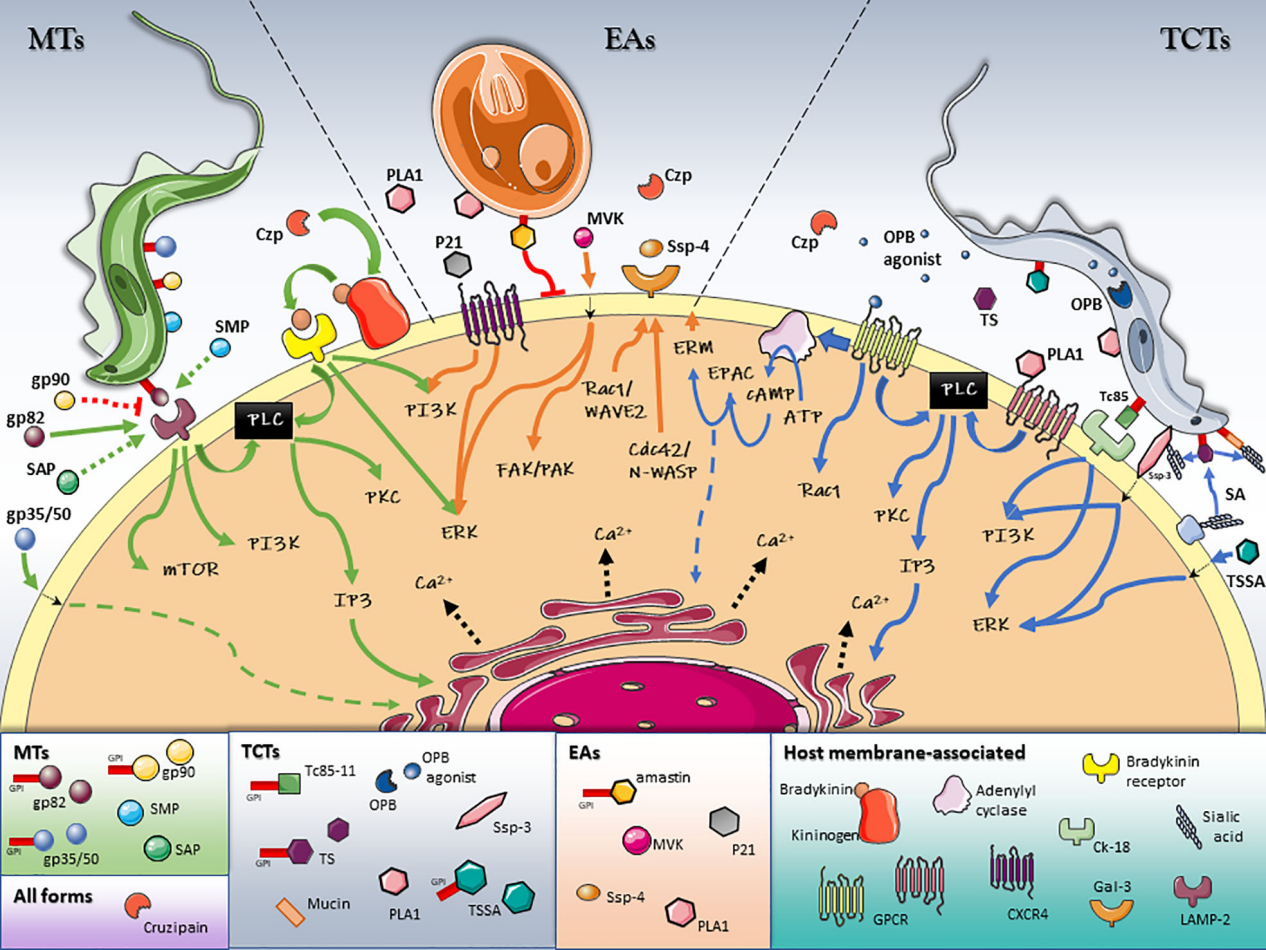
Schematic model of T. cruzi/host cell protein interactions and activated signaling pathways during invasion. Stage-specific Surface Molecules expressed on the membrane of the parasite or shed into the extracellular medium, play an essential role in the recognition, adhesion and activation of signaling pathways that lead to a successful invasion of the host cell. Figures were created using images from Servier Medical Art Commons Attribution 3.0 Unported License. (http://smart.servier.com). Servier Medical Art by Servier is licensed under a Creative Commons Attribution 3.0 Unported License.

Bidirectional signaling pathways are activated in both the parasite and the host cell during invasion. *T. cruzi* specific signal transduction pathways have recently been reviewed elsewhere (Schoijet et al., 2019). This review provides a general overview of the key parasite/host interactions and signaling pathways activated in the host cell during *T. cruzi* invasion, which are summarized in **Table 1** and **Figure 1**.

**Table 1:** Stage-specific proteins involved during invasion by *T. cruzi*.

Stage	Molecule	Surface, secreted, or both	Signaling	Function	Ref.
*Metacyclic trypomastigotes (MTs)*	gp82	Both	PLC, mTOR and PI3K	Ca^2+^ and lysosome mobilization	(Teixeira and Yoshida, 1986; Cortez et al., 2014)
	gp90	Both		Inhibit gp82-mediated internalization	(Cordero et al., 2008; Martins et al., 2009; Rodrigues et al., 2017)
	gp35/50	Both		Ca^2+^ elevation and actin cytoskeleton-dependent invasion	(Ramirez et al., 1993; Dorta et al., 1995; Ferreira et al., 2006)
	SAP	Secreted		Enhance gp82-mediated internalization	(Baida et al., 2006; Zanforlin et al., 2013)
	TcSMP	Both		Enhance gp82-mediated internalization	(Martins et al., 2015)
*Tissue-culture trypomastigotes (TCTs)*	TS and iTS	Both	PI3K/Akt and MAPK/Erk	Promotion of invasion and sialylation pattern	(Chuenkova et al., 2001; Butler et al., 2013; Campetella et al., 2020)
	Tc85	Surface	ERK1/2	Host cell attachment and invasion	(Magdesian et al., 2007; Mattos et al., 2014)
	TSSA	Surface	ERK1/2	Host cell attachment and Ca^2+^ signaling	(Cánepa et al., 2012a; Cámara et al., 2017)
	TcOPB	Secreted	PLC and Rac1	Produces an unknown structure soluble factor that triggers Ca^2+^ mobilization	(Caler et al., 1998; Motta et al., 2019)
*Extracellular amastigotes (EAs)*	Amastin	Surface		Inhibit cell invasion	(Cruz et al., 2012)
	P21	Secreted	ERK and PI3K	Phagocytosis and actin cytoskeleton remodeling	(Rodrigues et al., 2012; Teixeira T. L. et al., 2015)
	TcMVK	Secreted	P38/ERK and FAK/PAK	Protein glycosylation and cytoskeletal assembly	(Ferreira et al., 2016)
	Ssp-4	Secreted	Rac1/WAVE2 and Cdc42/N-WASP	Associated with host cell invasion	(Florentino et al., 2018)
*TCTs and EAs*	TcPLA1	Both	PKC	Lipid profile modification and amastigote development	(Wainszelbaum et al., 2001; Belaunzarán et al., 2007)
*All forms*	Cruzipain	Secreted	PI3K/Akt and MEK/ERK	Ca^2+^ signaling	(Taketo et al., 1997; San Francisco et al., 2017)

## The Prelude to Invasion: Host Cell Recognition, Adhesion, and Activation

### Stage-Specific Surface Molecules

Early studies have shown that proteolytic treatment of trypomastigotes resulted in 90% inhibition of invasion, establishing a clear correlation between parasite surface proteins and infectivity (Andrews et al., 1984). Since then, several surface proteins have been identified and characterized. More recently, the first mass spectrometry-based exhaustive glycoproteome analysis of T. cruzi was completed, allowing the identification of 690 glycoproteins. Among them, 170 were exclusively identified in epimastigotes and 334 in trypomastigotes (Alves et al., 2017). In addition, it has been well established that every infective form of *T. cruzi* (MTs, TCTs and EAs) expresses on its surface a distinct set of stage-specific glycoproteins (Alba Soto and González Cappa, 2019). Several parasite stage-specific glycoprotein/host cell receptor interactions, and the corresponding signaling cascade activated in the host, are currently known (**Figure 1** and **Table 1**).

#### Metacyclic Trypomastigotes

##### - gp82

Among surface glycoproteins involved in the adhesion of *T. cruzi* to the host cell is gp82, a MT-specific virulence factor, member of the gp85/TS family (Teixeira and Yoshida, 1986; Cortez et al., 2012; Maeda et al., 2012). Gp82 is attached to the outer cell membrane of the parasite by a glycosylphosphatidylinositol (GPI) anchor, which is susceptible to cleavage by an endogenous phosphatidylinositol-specific phospholipase C (PI-PLC) and released into the extracellular medium (Bayer-Santos et al., 2013a). During the MTs invasion process, secreted, and/or surface-anchored gp82 molecules interact with a host receptor and trigger signaling pathways leading to intracellular Ca^2+^ and lysosome mobilization in the host cell (Manque et al., 2003). Gp82 mediates the mobilization of Ca^2+^ from thapsigargin-sensitive intracellular stores (Yoshida et al., 2000). The activation of PLC in the host cell generats diacylglycerol (DAG) and inositol 1,4,5-trisphosphate (IP3), which induces protein kinase C (PKC) and promotes the release of Ca^2+^ from IP3-sensitive compartments (Maeda et al., 2012). Mammalian target of rapamycin (mTOR) and phosphatidylinositol 3-kinase (PI3K) are also activated during MTs invasion. The elevation in the cytosolic Ca^2+^ concentration triggered by these pathways, promotes actin cytoskeleton disruption and lysosome mobilization to the cell periphery, both events promoting the internalization of the parasite (Martins et al., 2011; Cortez et al., 2014). The lysosome-associated membrane protein 2 (LAMP‐2) has been recently identified as the host cell receptor for gp82 (Rodrigues et al., 2019). In this work, Rodrigues et al. have shown that antibodies directed against LAMP‐2, but not to LAMP‐1, significantly inhibit MTs internalization. Moreover, co‐immunoprecipitation assays demonstrated that gp82 binds to LAMP‐2 protein in a receptor‐mediated manner (Rodrigues et al., 2019).

##### - gp90

Another key MTs surface glycoprotein is gp90, also a member of the gp85/TS superfamily, which in opposition to gp82, has a negative effect on parasite invasion (Bubis et al., 2018). Early studies have shown an inverse correlation between gp90 expression levels and MTs infectivity (Málaga and Yoshida, 2001). Moreover, monoclonal antibodies confirmed low levels of gp90 in a highly invasive strain (CL), while high expression of gp90 was observed in a poor invasive strain (G) (Ruiz et al., 1998). Although gp90 binds to the host cell, it fails to trigger cytosolic Ca^2+^ mobilization (Ruiz et al., 1998). The inhibition of host cell lysosome spreading has been recently proposed as the mechanism by which gp90 exerts its down-regulatory role (Rodrigues et al., 2017).

##### - gp35/50

Gp35/50 mucins are highly glycosylated proteins expressed by MTs forms of *T. cruzi* (Ramirez et al., 1993). Like gp82, gp35/50 has the ability to trigger intracellular Ca^2+^ elevation when binding to host cell (Dorta et al., 1995; Ruiz et al., 1998). However, gp35/50-medited invasion induces actin recuitment, in contrast to gp82, that triggers signaling pathways leading to disassembly of F-actin (Ferreira et al., 2006). In addition, high level expression of gp35/50 was found to be inversely correlated to infectivity (Ruiz et al., 1998), although, when treatment of MTs with neuraminidase was applied before invasion assays, an increase in infectivity was observed, probably due to the fact that desialylated gp35/50 can interact with the host cell (Yoshida et al., 1997).

#### Cell-Derived Trypomastigotes

##### - Trans-sialidase

Among the different surface molecules involved in TCTs invasion is the unique *T. cruzi* trans-sialidase (TS), an important parasite virulence factor. Unable to synthesize sialic acid (SA), TS enables TCTs to transfer terminal SA residues linked α2,3 to terminal β-galactopyranoses from host cell donor macromolecules to glycans of mucin-type proteins displayed on the parasite membrane (Schenkman et al., 1991; da Fonseca et al., 2019; Campetella et al., 2020). The generation of a sialylated surface plays a central role in promoting the evasion of immune responses, favoring survival and the establishment of the chronic disease (Nardy et al., 2016). In addition, the transference of SA to the parasite surface creates the Stage-Specific Epitope 3 (Ssp-3) that promotes invasion of the host cell (Schenkman et al., 1991). TS has also been postulated as counter-receptor for TCTs binding to α2,3-sialyl receptors on the host cell, as a prelude to *T. cruzi* invasion (Ming et al., 1993). Signaling pathways implicated in TS mediated promotion of invasion includes the PI3K/AKT (Chuenkova et al., 2001; Butler et al., 2013) and the MAPK/ERK (mitogen-activated protein kinase/extracellular regulated kinase) pathways (Chuenkova and Pereira, 2001). Furthermore, shedding of TS into the bloodstream allows *T. cruzi* to manipulate the surface sialylation pattern of the target cell and different cell types distant from the site of infection. This soluble form of TS has been involved, among other processes, in host immunomodulation and haematological alterations, mainly by disruption of cell surface sialyl homeostasis (Campetella et al., 2020). Moreover, differential TS expression and gene dosage between different *T. cruzi* strains, have been reported. In a murine model, highly virulent strains of the parasite, belonging to DTU-VI, expressed and shed high amounts of TS, whereas the opposite was observed in mice infected by the low-virulence DTU-I strains (Risso et al., 2004; Burgos et al., 2013). Intriguingly, a naturally occurring point mutation, the Y342H substitution, accounts for the lack of trans-sialylation activity that generates an inactive form of TS (iTS) (Cremona et al., 1995). Still, iTS behaves as a lectin-like protein, that maintains the ability to bind SA and β-galactose residues (Cremona et al., 1999). Experimental data strongly suggest that iTS confers alternative and/or complementary roles to TS in the parasite virulence and pathogenesis (Campetella et al., 2020).

##### - Tc85

Another TCTs surface molecule with affinity for the extracellular matrix is the Tc85 family (Giordano et al., 1999). Belonging to the gp85/TS superfamily, Tc85 proteins lack enzymatic activity and, although unable to transfer SA, they have been implicated in cell adhesion and invasion (Mattos et al., 2014). A Laminin-G like domain (LamG) at the C-terminus of gp85/TS seems to be responsible for binding different receptors present in the extracellular matrix and host cell surface (Teixeira A. A. R. et al., 2015). Two motifs in the LamG domain have been described: The FLY motif (VTVxNVxLYNRPLN), present at the C-terminus of Tc85 proteins, mediates the interaction with cytokeratins (Tonelli et al., 2010), and the TS9 motif that showed significant cell binding capacity (Teixeira A. A. R. et al., 2015). In particular, FLY has been implicated in cytokeratin remodeling, ERK1/2 signaling pathway activation and increased internalization (Magdesian et al., 2007). It was shown that the FLY interacts with the endothelium in an organ-dependent manner with significantly higher avidity for the heart vasculature (Tonelli et al., 2010). These results, and the fact that TS9 and FLY are separated from each other by approximately 100 amino acids in the primary sequence of the gp85/TS proteins, are in agreement with the idea that TS9 and FLY comprise a non-linear conformational binding site (Teixeira A. A. R. et al., 2015).

##### - TSSA

The trypomastigote small surface antigen (TSSA) is a small mucin-like protein from the TcMUC family of *T. cruzi* mucin genes, the main mucins on the surface of TCTs and the scaffolds of the Ssp-3 epitope (Buscaglia et al., 2004; Campo et al., 2006). Although TSSA is not a SA acceptor, it binds to mammalian cells and induces Ca^2+^ signaling (Cánepa et al., 2012a). There are four allelic variants (TSSA I-IV), each one corresponding to an ancestral DTU (I-IV), while in hybrid genomes (DTU V-VI) TSSA isoforms II and III can be found (Balouz et al., 2021). TSSAII showed higher adhesion to host cells than TSSAI. Furthermore, TSSAII elicited a much more rapid and sustained increase in intracellular Ca^2+^ and promoted a stronger stimulation of the ERK1/2 pathway, than TSSA I (Cánepa et al., 2012a). Mapping experiments and cell-binding assays revealed that at least two peptidic motifs are critical for the interaction of the “adhesive” TSSA variant to host cell surface receptor(s) prior to trypomastigote internalization. These observations were supported by the fact that transgenic trypomastigotes over-expressing the ‘adhesive’ TSSA displayed improved adhesion and infectivity towards non-macrophagic cell lines (Cámara et al., 2017).

#### Extracellular Amastigotes

##### - δ-Amastin

The amastin multi-gene family was originally identified by screening an amastigote cDNA library (Teixeira et al., 1994). In particular, δ-Amastin, a transmembrane glycoprotein highly expressed on the surface of intracellular amastigotes, has been implicated in EAs cell invasion and differentiation (Cruz et al., 2012). Although amastin is present in all sequenced *T. cruzi* strains (Cerqueira et al., 2008), transcript levels were found to be down-regulated in amastigotes of the G strain (Cruz et al., 2012; Kangussu-Marcolino et al., 2013). It was shown that recombinant δ-amastin binds to cells in a saturable and dose-dependent manner and was able to inhibit parasite internalization, suggesting a role for amastin in *T. cruzi* invasion (Cruz et al., 2012). Moreover, in transgenic EAs, the overexpression of amastin promoted liver tropism during *in vivo* infections in mice and accelerated amastigogenesis (Cruz et al., 2012). The involvement of amastins in *T. cruzi* virulence was also supported by knocking down δ-amastins in *Leshmania braziliensis*, which resulted in a decrease in survival and proliferation of intracellular parasites after *in vitro* macrophage infection and no detectable parasites after *in vivo* infections (de Paiva et al., 2015).

#### Protein Secretion and Extracellular Vesicles Cargo

Trypomastigotes (MTs and TCTs) and EAs shed a wide number of GPI-anchored surface proteins/glycoproteins such as members of the gp85/TS family, mucins and MASPs (Trocoli Torrecilhas et al., 2009; Cánepa et al., 2012b; Bayer-Santos et al., 2013b; Lantos et al., 2016; Watanabe Costa et al., 2016). These proteins are not secreted by the classical endoplasmic reticulum (ER)/Golgi-dependent secretion pathway, but instead, gradually released into milieu by the action of an endogenous PI-PLC (Andrews et al., 1988), or associated to extracellular vesicles (EVs) involved in host cell invasion, immunomodulation and pathogenesis (Borges et al., 2016; Torrecilhas et al., 2020).

EVs can be divided into: microvesicles or ectosomes (100 nm to1 μM), directly originated by budding from plasma membrane, and exosomes (30–100 nM), that are secreted following the fusion of multivesicular endosomes with the membrane at the flagellar pocket (Evans-Osses et al., 2015). Quantitative proteomic analysis revealed differences in protein content between these two populations of EVs (Bayer-Santos et al., 2013a). An interesting case is the trypomastigote excreted/secreted antigens (TESA), around 80 parasite proteins with the majority being highly immunogenic gp85s, associated with exosomal vesicles shed by MTs, TCTs, and intracellular amastigotes, used as a reagent in the diagnosis of the disease (Berrizbeitia et al., 2006; Bautista-López et al., 2017). Although EVs are secreted by all forms of *T. cruzi*, only those shed by infective forms are able to enhance internalization of host cells, by inducing intracellular Ca^2+^ mobilization (Moreira et al., 2019). Inoculation of EVs before infection in mice produced an increment of parasitemia in early days post-infection and more amastigote nests in mice hearts (Lovo-Martins et al., 2018). Moreover, it has been shown that vesicles from TCTs from the *T. cruzi* strains Colombiana (DTU I), YuYu (DTU I), Y (DTU II), and CL-14 (DTU VI) presented differences in their protein and α-galactosyl contents and were able to differentially modulate host’s immune responses and parasite invasion (Nogueira et al., 2015). Although all strains were capable of activating MAPKs like p38, JNK, and ERK 1/2, CL-14, and YuYu activated MAPKs *via* TLR2, while EVs from Colombiana and Y strains needed to be internalized to activate the MAPK pathway (Nogueira et al., 2015). Thus, the composition and effects of EVs on host cell seems to be strain-dependent.

In addition to glycoproteins, a substantial number of other molecules are released into the extracellular medium, like complement regulatory proteins (CRPs), cruzipain (Czp), peptidyl-prolyl cis-trans-isomerases, oligopeptidases and proteases, phospholipases A1 and C, P21, and amastigote specific proteins (Torrecilhas et al., 2012; Watanabe Costa et al., 2016).

Interesting examples are SAP (serine-, alanine-, and proline-rich protein) and TcSMP (*Trypanosoma cruzi* Surface Membrane Proteins), which have been involved in MTs invasion by binding to host cells and triggering Ca^2+^ signaling and lysosome mobilization (Baida et al., 2006; Zanforlin et al., 2013; Martins et al., 2015).

##### - SAP

Diverse paralogs of SAPs, with different cellular localization, are expressed in the different development stages of the parasite. In particular, SAP peptides were identified by by mass spectrometry in vesicle and soluble-protein fractions from epimastigotes and MTs conditioned medium. Although, SAP transcript levels and protein expression in MTs were found to be twice as high as in epimastigotes, in agreement with their proposed role in cell adhesion and invasion (Zanforlin et al., 2013). In this regard, the fact that gp82 and SAP share the ability to induce Ca^2+^ signaling and lysosome mobilization, led to the hypothesis that both molecules display a synergistic effect in the process of MTs host-cell invasion (Baida et al., 2006; Zanforlin et al., 2013).

##### - TcSMP

Recently described, the TcSMP family, possesses two main features typical of surface proteins, an N-terminal signal peptide and a C-terminal hydrophobic sequence, predicted to be a transmembrane domain, rather than the most prevalent GPI anchoring (Martins et al., 2015). TcSMPs are expressed in all *T. cruzi* developmental stages, located at the surface and present in the secretome of epimastigotes and MTs. Similarly to SAP, TcSMPs have been shown to promote a weaker lysosome mobilization and parasite internalization than gp82, suggesting an auxiliary role in parasite invasion (Martins et al., 2015).

##### - TcPLA1

The membrane-associated phospholipase A1 (TcPLA1) can be also found in the extracellular medium of TCTs and EAs (Belaunzarán et al., 2007). Host cells exposed to the conditioned medium of EAs, TCTs, or recombinant TcPLA1, showed modified lipid profiles, with increased cellular concentrations of free fatty acids, diacylglycerol and lysophosphatidylcholine, that contributed to the concomitant activation of the PKC pathway (Belaunzarán et al., 2013). Remarkably, PKC has been previously implicated in parasite invasion, suggesting that Tc-PLA1 would participate in the events preceding host cell invasion (Watanabe Costa et al., 2016).

#### Peptidases

Peptidases, a class of hydrolytic enzymes responsible for breaking peptide bonds, has attracted the attention of distinct research groups because of their role in several crucial steps of the life cycle of the trypanosomatid parasites. The *T. cruzi* genome contains several families of peptidases that play central roles in diverse processes, such as adhesion and cell invasion (Alvarez et al., 2012; Rawlings et al., 2014).

##### - Cruzipain

Cruzipain (Czp), the most notorious cysteine peptidase, is expressed as a complex mixture of isoforms in all forms of *T. cruzi* and mainly located in lysosome-related organelles (Lima et al., 2012), have been shown to be required but not essential for invasion (San Francisco et al., 2017). Czp released by trypomastigote promotes invasion through its cysteine protease activity by producing bradykinin from membrane-bound kininogen on the surface of the host cell and triggering IP3-mediated Ca^2+^ signaling upon recognition by bradykinin B2 receptor (B_2_R) (Scharfstein et al., 2000). More recently, a second cruzipain-mediated route, blocked by a cysteine protease inhibitor, thapsigargin and immunodepletion of Czp, but not by kinin receptor antagonists, was described for TCTs (Aparicio et al., 2004). Experimental data evidenced that this effect is mediated by a soluble trypomastigote-associated factor released by Czp (Aparicio et al., 2004).

##### - Oligopeptidase B

Oligopeptidase B (OPB), a serine endopeptidase from the prolyl oligopeptidase family, is conserved in trypanosomatids but not present in any mammalian genome (Motta et al., 2019). OPB has a cytosolic localization and there is not any strong evidence suggesting its secretion by the parasite. Instead, it has been involved in the cytoplasmatic processing of a trypomastigote-specific precursor that generates a soluble factor of unknown structure which is shed by TCTs (Burleigh et al., 1997).

Upon binding to the host cell receptor, the OPB-agonist induces PLC activation and an IP3-dependent release of Ca^2+^ from intracellular stores. This Ca^2+^ mobilization promotes lysosomal recruitment to the entry site and F-actin filaments disruption, both events associated with an increased parasite invasion (Burleigh et al., 1997; Caler et al., 1998). Surprisingly, even today, with genomes of several *T. cruzi* strains available, the identity of the precursor it is still unknown. However, the secretion of OPB cannot be ruled out since OPB activity has been found in trypomastigotes supernatants (Fernandes et al., 2005; Motta et al., 2019). *Trypanosoma brucei* and *Trypanosoma evansi* OPBs, are released into the extracellular milieu and contribute to pathogenesis by hydrolyzing host circulating factors (Motta et al., 2019). In the case of *T. cruzi*, hydrolyzed peptides would mimics ligands capable of activating GPCR and/or RTK (Motta et al., 2019).

#### EAs Specific Proteins

EAs are capable of invading mammalian cells in an actin-dependent mechanism, forming a phagocytic cup that engulfs the parasite (Mortara et al., 2005). Secreted proteins from EAs, such as P21, mevalonate kinase (TcMVK) and specific-surface protein 4 (Ssp-4), mediate host cell signaling during the phagocytosis-like mechanism of invasion (Rodrigues et al., 2012; Ferreira et al., 2016; Florentino et al., 2018).

##### - P21

P21 is a 21kDa protein expressed in all developmental stages of *T. cruzi* and secreted by EAs to induce host cell invasion (da Silva et al., 2009). Evidence for this observation came from the use of a recombinant version of P21 (rP21) that bound to the CXCR4 chemokine receptor and promoted phagocytosis by induction of actin cytoskeleton polymerization and the modulation of the expression of actin-related genes in a PI3K-dependent manner (Rodrigues et al., 2012; Teixeira et al., 2017). In addition, in mice infections with the *T. cruzi* naturally attenuated TCC strain, rP21 lead to an exacerbated infection and parasite load in target organs (Brandán et al., 2019).

##### - TcMVK

MVK is a key enzyme involved in the early steps of the sterol isoprenoids biosynthesis pathway (Ferreira et al., 2016). In T. cruzi, TcMVK localizes to glycosomes, and may be also secreted into the extracellular milieu where it modulate host cell invasion, independently of its catalytic function. More precisely, TcMVK activates the actin-related kinases FAK (focal adhesion kinase) and PAK (p21-activated kinase), and the MAPK pathway components, ERK, and p38 to promote EAs internalization (Ferreira et al., 2016).

##### - Ssp-4

Ssp4 is a major surface GPI-anchored glycoprotein that is secreted by the EAs (Andrews et al., 1987). Although EAs Ssp-4 expression does not correlate with infectivity, glycosylation of Ssp-4 was associated with host cell invasion. It has been shown that only EAs from highly infective strains secreted a differentially glycosylated Ssp-4 into vesicle trails at the site of entry, contributing to Galectin-3 (Gal-3) recruitment and establishing a physical link between the parasite and the host cell surface (Florentino et al., 2018). Gal-3, a 31kDa β-galactoside-binding protein, is recruited to the site of EAs entry during cell invasion and participates in the intracellular trafficking of the parasite (Machado et al., 2014).

### Plasma Membrane Damage

It has been proposed that flagellar motility of trypomastigotes strongly attached to the host cells surface through their posterior end produces membrane damage in the host cell. An active gliding motility of parasites firmly attached to host cells was evidenced using time-lapse phase-contrast live images of trypomastigotes interacting with a HeLa cells (Fernandes et al., 2011). Supporting evidence was also obtained from the analysis of scanning electron microscopy images of *T. cruzi* during early steps of invasion, showing parasites gliding under cells or in close contact with the plasma membrane at the cell periphery (Fernandes et al., 2011). Parasite-mediated membrane damage triggers Ca^2+^-dependent fusion of lysosomes and internalization through Plasma Membrane Repair Mechanism (PMR), that will be discussed below.

## Hijacking Host’s Signaling Machinery

**Figure 2:**
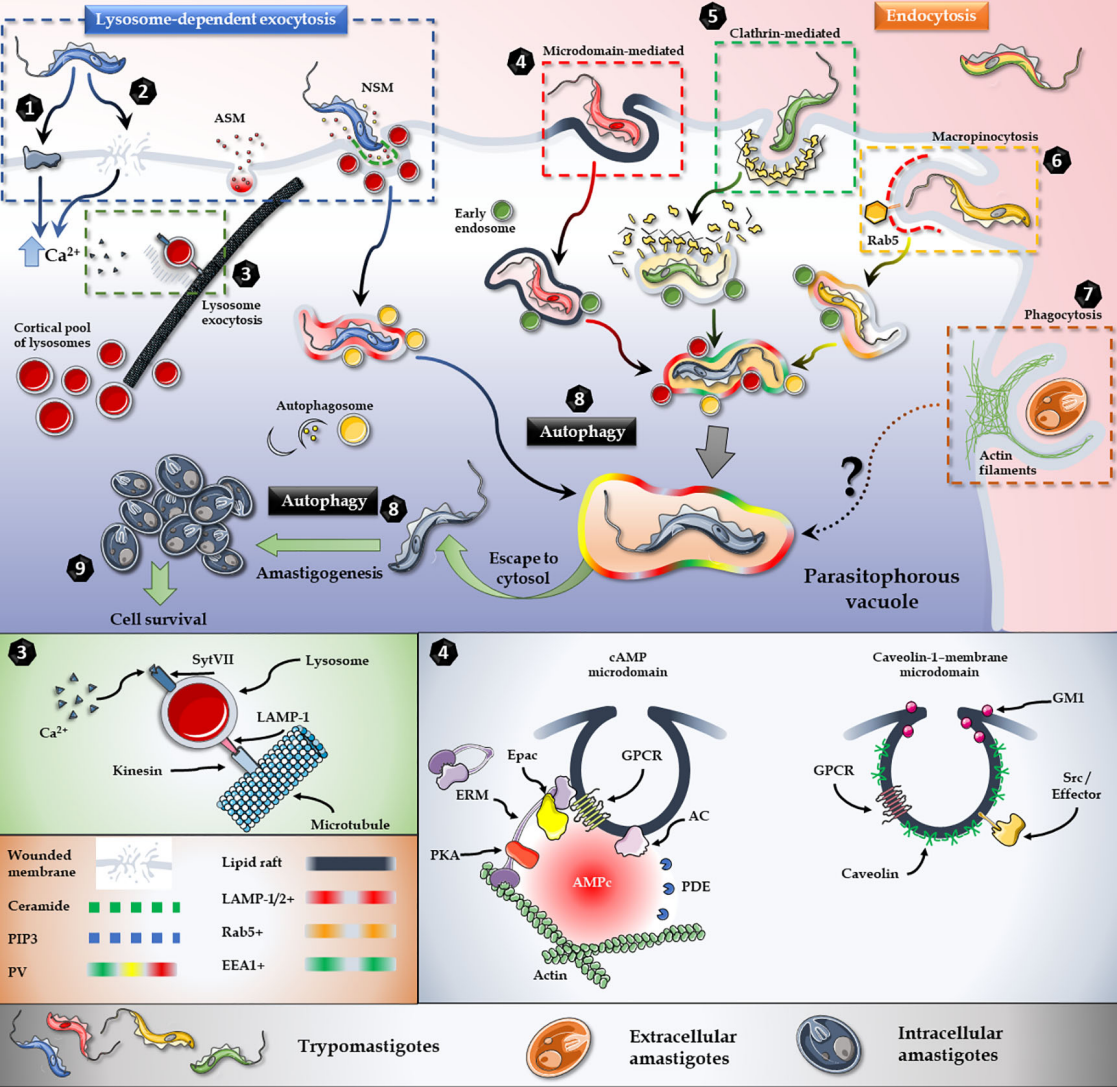
T. cruzi invasion model. Lysosome exocytosis involves surface/secreted proteins (1) or micro-injuries (2) that trigger an elevation in the intracellular levels of Ca2+ and the microtubule/kinesin-mediated recruitment of lysosomes from surrounding areas to the parasite entry site (3). Host’s Acid Sphingomyelinase (ASM) and a parasite Neutral Sphingomyelinase (NSM) are secreted to the extracellular milieu and participates in the breakdown of sphingomyelin to ceramide and phosphorylcholine in the outer leaflet of the plasma membrane (3). Endocytic mechanisms, such as lipid raft-dependent endocytosis (4), clathrin-mediated endocytosis (5) and macropinocytosis (6) also converge in the internalization of the parasite. Extracellular amastigotes, on the other hand, employ a phagocytosis-like mechanism for invasion (7). Moreover, autophagy is a key player during the invasion and also promotes trypomastigotes to amastigotes differentiation (8). In brief, regardless of the parasite stage, or the activated cascade, all internalization pathways culminate in the parasitophorous vacuole, from which parasite escapes to the cytoplasm and differentiates into amastigotes and proliferates (9). Figures were created using images from Servier Medical Art Commons Attribution 3.0 Unported License. (http://smart.servier.com). Servier Medical Art by Servier is licensed under a Creative Commons Attribution 3.0 Unported License.

To maintain homeostasis, host cells have a complex vesicular transport system, that consist of multiple connected networks with different levels of cross-talk (Salimi et al., 2020). *T. cruzi* has developed the ability of subverting and exploiting the most suitable mechanism at the time of invasion to gain access to the host cell (**Figure 2**). Three main mechanisms of internalization, involving several coordinated and integrated pathways, are used by the parasite to gain access to the target cell: 1) Ca^2+^-mediated recruitment and fusion of lysosomes to the entry site (**Figures 2.1–3**), 2) Endocytosis of plasma membrane (**Figures 2.4–7**) and 3) Autophagy (**Figure 2.8**).

It is important to note that regardless of the parasite strain, the developmental stage, the repertoire of surface/secreted molecules expressed and the signaling cascades activated to prepare the host cell for invasion, all the internalization mechanisms lead to the biogenesis of TcPV (Batista et al., 2020). Moreover, despite the invasion mechanism as well, the acquisition of lysosome markers by the TcPV during the process of internalization has shown to be essential for intracellular retention of the parasite and the establishment of a successful infection (Andrade and Andrews, 2004).

### Plasma Membrane Repair Mechanism

In order to maintain cellular homeostasis, membrane disruptions are rapidly resealed by a conserved PMR. Upon wounding, toxic levels of Ca^2+^ and oxidants from the extracellular milieu enter the cell. To avoid cell death, the damage is rapidly repaired by an extracellular Ca^2+^-induced recruitment of intracellular vesicles (Blazek et al., 2015) (**Figures 2.1–3**). It has been shown that conventional lysosomal exocytosis mediates the resealing in primary skin fibroblasts (Reddy et al., 2001). After wounding, Ca^2+^-dependent fusion of lysosomes to the host cell membrane was evidenced by the exposure of the luminal domain of lysosomal LAMP-1 and Synaptotagmin isoform VII (SytVII), a putative Ca^2+^ sensor in exocytosis (Sugita et al., 2001).

Moreover, a SytVII regulation of lysosome Ca^2+^-dependent exocytosis was evident by a dramatic inhibitory effect on plasma membrane resealing by antibodies directed against the cytosolic domain of this protein (Reddy et al., 2001). In addition to the exposure of lysosomal luminal proteins on the surface of the cell, the lysosomal enzyme Acid Sphingomyelinase (ASM) is secreted during cell injury and promotes plasma membrane repair (Tam et al., 2010) (**Figures 2.1–3**).

Invasion assays in the presence the pore-forming bacterial toxin Streptolysin O (SLO) increased parasite internalization, while bromoenol lactone (BEL), a lysosomal exocytosis inhibitor, strongly restrained invasion by *T. cruzi* trypomastigotes. Moreover, PMR and *T. cruzi* internalization have been shown to depend on the secretion of ASM, the lysosomal enzyme responsible for catalysing the breakdown of sphingomyelin to ceramide and phosphorylcholine in the outer leaflet of the plasma membrane (Tam et al., 2010; Fernandes et al., 2011). Lysosomal cysteine proteases cathepsin B and L are also secreted and may participate in the repair process by facilitating membrane access of ASM (Castro-Gomes et al., 2016). As a result, surface staining with anti-ceramide monoclonal antibodies and EEA1(early endosome-associated protein)-positive vesicles increased after treatement with extracellular ASM, suggesting that ceramide-enriched endocytic vesicles formation can facilitate trypomastigote entry. In the same line, inhibition of ASM reduced trypomastigote entry and this inhibition was reverted by the addition of extracellular sphingomyelinase (Fernandes et al., 2011). Proteomics studies have shown that trypomastigotes express and shed a neutral sphingomyelinase (Brossas et al., 2017) that might be contributing to the production of the required ceramide in the outer leaflet of the plasma membrane during host cell invasion by *T. cruzi*, although this hypothesis has not been yet explored. In accordance with the PMR-mediated invasion model, it was recently reported that the parasite could modulate the expression of plasma membrane repair-related proteins and the fold of change depends on the number of parasites interacting with the host cell (Brígido et al., 2017) (**Figures 2.1–3**).

### Ca^2+^-Dependent Lysosome Exocytosis

Originally considered terminal degradative organelles, lysosomes have been found to participate in many other cellular processes (Pu et al., 2016). The involvement of lysosomes in these different processes depends on their sub-cellular distribution and their ability to move throughout the cytoplasm (Pu et al., 2016). Lysosomes distribute in a rather immobile perinuclear pool and a more dynamic pool in the cell periphery (Cabukusta and Neefjes, 2018). Living cells video microscopy during TCTs host cell invasion showed a directional microtubule/kinesin-mediated migration of lysosomes from surrounding areas to the parasite entry site (Rodríguez et al., 1996). It was later demonstrated that TCTs uses the cortical pool of lysosomes in the invasion process (Hissa and Andrade, 2015) (**Figures 2.1–3**).

Membrane-associated rafts enriched in cholesterol and ganglioside GM1 have been also implicated in adhesion and internalization of all infective forms of *T. cruzi* (Barrias et al., 2007; Fernandes et al., 2007). Immunofluorescence analysis demonstrated a colocalization of GM1, flotillin 1, and caveolin 1 in the nascent TcPV, supporting the fact that membrane rafts participate in *T. cruzi* invasion (Barrias et al., 2007). Consistently, cholesterol involvement in the recruitment of lysosomes was evidenced using methyl-beta cyclodextrin (MβCD), a cholesterol-removing agent used for lipid raft disruption. Noteworthy, LAMP-2 have shown to play a major role in cholesterol and caveolin traffic, membrane repair and *T. cruzi* invasion. Cells lacking LAMP-2 showed deficiency in cholesterol delivered to the plasma membrane and an altered caveolin-1 distribution, both phenomena being refractory to TCTs invasion (do Couto et al., 2020). Similarly, MTs internalization was significantly reduced in LAMP-2-depleted HeLa cells (Cortez et al., 2016).

In MTs invasion of Hela cells, lysosome biogenesis/scattering was stimulated upon interaction of the parasites with the host cell and a reduction in the number of cortical lysosomes negatively affected MTs invasion (Cortez et al., 2016), as previously reported for TCTs invasion of cardiomyocytes (Hissa et al., 2012; Hissa and Andrade, 2015). However, in the HeLa model, the stimulation of lysosome biogenesis/scattering diminished TCTs ability for invasion, whereas rapamycin-promoted lysosome accumulation at the perinuclear region led to a higher TCTs invasion (Cortez et al., 2016). While these observations may result contradictory, it is important to consider that different parasite strains have been used in order to establish the different models of invasion. *T. cruzi* invasion, already showed to be a complex process when only taking into account the different stages of the invading parasite. This complexity gets even higher when considering different DTUs, strains, the repertoire of surface/secreted molecules, and signaling pathways activated in the host cell. In this regard, differential infectivity has been reported for trypomastigotes of different strains (Cortez et al., 2012; Santi-Rocca et al., 2017); thus, it is not unlikely that a particular mechanism of invasion is exploited depending on the strain and host cell/tissue. For example, in contrast to the results reported for TCTs, the absence of extracellular Ca^2+^ had no effect on MTs invasion, while the presence of the pore‐forming bacterial toxin SLO decreased MTs internalization (Rodrigues et al., 2019), strongly suggesting a PMR-independent mechanism of invasion for MTs.

Ca^2+^ release from cellular compartments, such as the endoplasmic reticulum, is accompanied by an activation of PLC and an elevation of intracellular cAMP levels. It has been shown that cAMP is able to potentiate the Ca^2+^-dependent exocytosis of lysosomes and lysosome-mediated cell invasion by *T. cruzi* (Rodríguez et al., 1999). In mammalian cells, both cAMP effector pathways, i.e., Protein Kinase A (PKA) and Exchange protein activated directly by cAMP (Epac), are involved in Ca^2+^-triggered exocytic events (Seino and Shibasaki, 2005). Moreover, members of this latter pathway, including Rap1, have been localized to late endosomes/lysosomes (Pizon et al., 1994), and Epac-mediated Rap activation has been involved in regulated exocytosis in human sperm (Miro-Moran et al., 2012), insulin secretion (Tengholm and Gylfe, 2017) and pancreatic amylase release (Sabbatini et al., 2008). Accordingly, it was recently shown that Epac1-mediated signaling represents the main mechanism for cAMP-dependent host cell invasion by *T. cruzi* (Musikant et al., 2017). Additionally, ERM proteins (ezrin, radixin and moesin), which are essential for the cell cortex function and architecture by linking plasma membrane to the underneath actin cytoskeleton (McClatchey, 2014), have been associated to *T. cruzi* invasion (Ferreira et al., 2017). Confocal microscopy studies have shown that ERM proteins are recruited to the EA invasion site, where they co-localize with F-actin, and that depletion of host ERM proteins inhibited T. cruzi invasion in HeLa cells (Ferreira et al., 2017). Remarkably, radixin was identified as a scaffolding unit for cAMP effectors in the spatial regulation of cAMP-Epac1-Rap-mediated signaling (Gloerich et al., 2010; Hochbaum et al., 2011). A link between radixin and cAMP-Epac-mediated TCTs invasion was recently evidenced by blocking invasion in pre-treated NRK host cells with a 15 amino acid permeable peptide spanning Epac’s minimal ERM-biding domain (Musikant et al., 2017). This observation was consistent with a co-localization of a pool of Epac1 and radixin, as a requirement for invasion. Also, F-actin regulation is in part due to the activity of the focal adhesion kinase (FAK), a cytoplasmic protein tyrosine kinase (PTK) that participates during invasion by *T. cruzi* (Melo et al., 2014) (**Figure 2.4**). Both, inhibition of FAK autophosphorylation or knockdown of FAK expression by siRNA in cardiomyocytes, led to a reduction in *T. cruzi* internalization, hence showing a key role of the FAK-mediated pathway in this process (Melo et al., 2014). FAK inhibition was associated with ERK1/2 dephosphorylation and F-actin rearrangement, suggesting a crosstalk between this signaling cascade and the MEK/ERK pathway (Onofre et al., 2019). Likewise, the interaction between HeLa cells and EAs induced N-WASP-dependent actin polymerization *via* PI3K/AKT and ERK but not SFK (Src family kinases) (Bonfim-Melo et al., 2018a). In opposition, previous works on cardiomyocytes have shown that Src was required for TCTs internalization (Melo et al., 2014). However, observations are not conclusive since internalization experiments were done using PP1 as Src inhibitor, which also blocks TGF-β-mediated cellular responses in a Src-independent fashion (Ming et al., 1995; Yoshida and Cortez, 2008; Ferrão et al., 2015; Silva et al., 2019).

#### Endocytic Pathways

Endocytic processes can be divided into different classes: clathrin-mediated, caveolae-mediated, membrane microdomain-mediated, macropinocytosis and phagocytosis (Chou et al., 2011). Several of these endocytic pathways are exploited by *T. cruzi* for invasion (Barrias et al., 2013) (**Figures 2.4–7**). Moreover, lysosome-independent endocytosis has been proposed to be the main entry mechanism for TCTs (Burleigh, 2005; Cortez et al., 2016).

##### - Phagocytosis

Experimental evidence showed that approximately 20 to 25% of the internalized trypomastigotes were associated to lysosomes, while 50% of the invading parasites exploited an alternative PI3-kinase-dependent mechanism of invasion, involving a host cell plasma membrane-derived vacuole enriched in the lipid products of class I PI3-kinases, Phosphatidylinositol 3-Phosphate PI3P/Phosphatidylinositol 3,4-bisphosphate (P3,4P2) (Woolsey et al., 2003). In this endocytic mechanism of internalization, downstream of cell entry the EEA1 marker was never associated to the parasite-containing vacuoles, instead, a gradual lysosomal fusion was revealed by the acquisition of lysosomal markers such as LAMP-1 and fluid-phase endocytic tracers from the lysosomal compartment (Woolsey et al., 2003). However, Rab5, a marker for early endosomes, was found to associate to a fraction of *T. cruzi*-containing vacuoles during and immediately following internalization. Likewise, the remaining 20% to 30% of the *T. cruzi*-containing vacuoles were positive for EEA1, the Rab5 effector, indicating that early endocytic pathway of internalization took place as well. Interestingly, it has been shown that the Toll‐Like Receptor 2 (TLR2) was required to activate PI3K and Rab5 binding to early endosomes in the Rab5/Rab7-endosome-dependent invasion mechanism (Maganto-Garcia et al., 2008). Accordingly, a strong activation of PI3K and PKB/AKT was detected when cells were incubated with trypomastigotes or their isolated membranes (Wilkowsky et al., 2001). Noteworthy, *T. cruzi*-infected human macrophages shed EVs that enhance host cell TLR-2-mediated invasion (Cronemberger-Andrade et al., 2020).

EAs employ a phagocytosis-like mechanism when invading non-professional phagocytic cells (Fernandes et al., 2013), with positive participation of Cdc42, N-WASP, WAVE2, and Rac1, and negative regulation of RhoA (Bonfim-Melo et al., 2018b) (**Figure 2.7**). Furthermore, EAs interaction with HeLa cells produced an increase in ERK1/2 phosphorylation, while pre-treatment of HeLa cells with an ERK1/2 inhibitor had a negative effect on internalization. This results demonstrated a key role for that PI3K/AKT and ERK pathway during *T. cruzi* EA invasion (Ferreira et al., 2019), probably through activation of proteins that regulate microfilament remodelling such as calpain, FAK and cortactin (Bonfim-Melo et al., 2018a).

##### - Clathrin-Mediated Endocytosis

Clathrin-mediated endocytosis in *T. cruzi* internalization was recently evidenced (Barrias et al., 2019) (**Figure 2.5**). Clathrin-containing vesicles and actin filaments were localized at sites of parasites attachment and internalization and around the nascent TcPV. Accordingly, specific inhibition of clathrin-coated pit formation impaired *T. cruzi* internalization (Barrias et al., 2019).

##### - Macropinocytosis

Macropinocytosis, an actin-driven process originally described as a mechanism of non-specific uptake of fluid into large cytoplasmic vesicles, has also been implicated in host cell invasion by *T. cruzi* is (King and Kay, 2019) (**Figure 2.6**). The nascent macropinosome accumulates PI3P and active Rab5, that regulates the fusion of membranous organelles at early stages of endocytosis (Feliciano et al., 2011). Signaling patches involving PIP3, Ras, and Rac direct actin polymerization to the periphery of the macropinocytic cup (Kay et al., 2018). The involvement of macropinocytosis as a mechanism of entry for *T. cruzi* was demonstrated by blocking parasite internalization using macropinocytosis inhibitors, such as amiloride, rottlerin and IPA3 (Barrias et al., 2012). In accordance, the stimulation of macropinocytic activity through activation of PKC by PMA, showed an increased internalization of parasites (Barrias et al., 2012). Moreover, colocalization at entry sites of trypomastigotes with the Rab5 effector rabankyrin 5, tyrosine kinases, Pak1 and actin microfilaments, confirmed macropinosomes formation (Barrias et al., 2012).

### Autophagic Pathway

Autophagy as an alternative pathway of internalization for *T. cruzi* was evidenced in starved cells, where the induction of autophagy was a positive modulator of invasion (**Figure 2.8**). On the other hand, the disruption of mammalian autophagy led to a reduction in infectivity (Salassa and Romano, 2019). The autophagy pathway consists of several coordinated and consecutive events: initiation, elongation, maturation, and fusion of lysosomes to the autophagosome. Upon activation, autophagosome biogenesis is initiated with the induction and nucleation of the phagophore, a double-membrane structure that grows to engulf the autophagic cargo, and the recruitment of the core autophagymachinery (Dikic and Elazar, 2018). Lipidated LC3-II is required in autophagosome biogenesis, and since it forms a stable association with the membrane of autophagosomes it is used as a marker for autophagy (Tanida et al., 2008). The presence of LC3 in the membrane of the TcPV during the internalization process showed a connection between the TCTs and the host-cell autophagic pathway (Romano et al., 2009). Accordingly, infection was reduced in the absence of specific autophagy genes Atg5 or Beclin1, confirming the requirement of an autophagic-derived compartment in autophagy-mediated invasion (Romano et al., 2009). Moreover, starvation and rapamycin treatment induced an increase of LAMP-1 in *T. cruzi*-containing vesicles, indicating lysosomal association to TcPV and the consequent autolysosome formation were required for an increased internalization (Romano et al., 2009).

## TcPV Maturation and Escape to Cytosol

Several proteins are recruited to the TcPV at different times during the biogenesis and maturation process (Batista et al., 2020). Within these proteins are the SNAREs, fusion proteins that regulate docking of granules and vesicles to target membranes including the plasma membrane (Wang et al., 2017). Vesicle associated membrane proteins 3 (VAMP3) and VAMP7, are consequtively recruited to the TcPV. VAMP3, usually present in recycling or early endosomes, is not essential for invasion, whereas SNARE complexes involving VAMP7, required for late endosome/lysosome fusion, are crucial in the establishment of *T. cruzi* infection (Cueto et al., 2017). Besides, early (Rab5, Rab22a, and Rab21 positive vesicles) and late (Rab7 and Rab39a) endocytic compartments, also recruited to the TcPV at early times post internalization, regulate the transit of the TcPV and promote fusion with lysosomes (Salassa et al., 2020). TcPV maturation is characterized by an initial interaction with Rab5 and VAMP3‐positive vesicles, followed by the recruitment of Rab7 and VAMP7, to finally fuse with lysosomes (Cueto et al., 2017; Salassa et al., 2020).

It is well established that fusion of lysosomes to the TcPV induces acidification that triggers the vacuole disruption and subsequent release of *T. cruzi* into the host cell cytosol (Ley et al., 1990). *T. cruzi* viability in the TcPV depends on a highly effective antioxidant defense machinery involving specialized antioxidant enzymes, such as peroxidases and superoxide dismutases (SODs), that protects the parasite against reactive oxygen and nitrogen species (Cardoso et al., 2016). Interestingly, oxidative stress has been shown to be an enhancer of *T. cruzi* infection in macrophages (Paiva et al., 2012). Although, a plausible hypothesis is that *T. cruzi* needs minimal levels of ROS, signaling for replication, while high levels of ROS are deleterious (Goes et al., 2016). Heavily sialylated LAMP-1 and 2, located in the inner coat of the TcPV, have been shown to protect the TcPV from lysis (Rubin-de-Celis et al., 2006). Additionally, LAMP-1 and 2 are essential to retain the intracellular parasite (Albertti et al., 2010) and avoid reversible invasion (Caradonna and Burleigh, 2011).

Under acidic conditions, the disruption of de TcPV occurs through the several *T. cruzi* proteins, such as secreted TS (Hall et al., 1992) and two pore-forming proteins, TcTOX (Andrews et al., 1990) and LYT1 (Manning-Cela et al., 2001). In TCTs, the expression of TS induces the escape from the TcPV by desialylation of LAMP-1 and 2, making membranes more susceptible to disruption by pore-forming proteins (Hall et al., 1992; Rubin-de-Celis et al., 2006). Pores are then formed by TcTOX and LYT1. Interestingly, TcTOX and LYT1 share similar characteristics: both are secreted, present cross-reactivity with C9 antibodies and have hemolytic activity at low PH. In fact, the molecular identity of TcTOX still remains unknown, and all available data suggest that LYT1 is TcTOX, or a TcTOX‐like protein (Benabdellah et al., 2007; Friedrich et al., 2012).

Once in the cytoplasm, host cellular and metabolic pathways will be targeted by amastigotes in order to successfully replicate. Four to seven days post-invasion, amastigotes differentiate into the non-replicative infective trypomastigote form, that is released into the bloodstream (Caradonna et al., 2013; Li et al., 2016; Oliveira et al., 2020)

## Concluding Remarks

Numerous works have endeavored to comprehend the molecular basis of *T. cruzi* invasion. Although some mechanisms involved in parasite/host interaction have been already described, a thorough understanding off the process would contribute to find new key players and provide a more diverse set of potential molecular targets against the disease. However, the evolution of this parasite has provided it with redundant and diverse molecular tools, able to interfere with multiple host cell pathways, to achieve a successful invasion.

The process of invasion begins with the recognition and adhesion of the parasite to the target cell. This interaction, reinforced by EVs and secreted proteins, leads to the activation of signaling pathways in the host cell that promote parasite internalization into an encasing vacuole, from which the parasite escapes to the cytosol where differentiation and replication take place.

Invasion has a pyramidal structure, in the base the diverse parasite/host protein interactions involved in internalization converge in the activation of a smaller set of signaling cascades and, regardless of the parasite strategy of internalization, all pathways end at the top of the pyramid with a parasite-containing vesicle to which lysosomes fuse to generate the parasitophorous vacuole. Little is known about the events that occur after TcPV is established, and a better understanding of this crucial mechanism may be the key to define new therapeutic targets against Chagas disease.

In this review, we address the several strategies *Trypanosoma cruzi*, the etiological agent of Chagas disease, has developed to subvert the host cell signaling pathways in order to gain access to the host cell cytoplasm, where replication and differentiation. Special attention is made to the numerous parasite/host protein interactions and the set of signaling cascades interfered during the formation of the parasitophorous vacuole. We first discuss the three strategies that *T. cruzi* exploits to trigger host cell signaling pathways to facilitate invasion: 1) Parasite surface/secreted proteins/host cell receptor interactions, 2) Protein shedding and 3) Host plasma membrane wounding. Later, strategies that lead to the internalization of the parasite, involving three main mechanisms: 1) Ca2+-dependent recruitment of lysosomes, 2) Endocytosis, and 3) Autophagy, are discussed. Finally, we examine the mechanisms by which the parasite escapes from the parasitophorous vacuole to establish a successful invasion. The topics discussed in this work were partially covered by other authors, however, we present a bigger picture, describing the complexity of the process considering genetic variability, strains, parasite/host interactions, signaling pathways activated and host cell. To our knowledge, this would be the more complete and updated review currently available.

## Author Contributions

GF and MME conceived, designed, and wrote the article. All authors contributed to the article and approved the submitted version.

## Funding

This work was partially supported by the FIC-NIH award number R03TW009001.

## Conflict of Interest

The authors declare that the research was conducted in the absence of any commercial or financial relationships that could be construed as a potential conflict of interest
